# Innate immune activation in vitiligo: mechanisms and pathophysiological implications

**DOI:** 10.3389/fimmu.2025.1631074

**Published:** 2025-10-15

**Authors:** Yuting Wang, Junhua Cao, Xiaomin Liu, Chengcheng Xiong, Da Xu, Fang Bian

**Affiliations:** ^1^ Department of Pharmacy, Xiangyang Central Hospital, Affiliated Hospital of Hubei University of Arts and Science, Xiangyang Key Laboratory of Special Preparation of Vitiligo, Xiangyang, China; ^2^ Central Laboratory, Xiangyang Central Hospital, Affiliated Hospital of Hubei University of Arts and Science, Xiangyang, China

**Keywords:** vitiligo, innate immunity, adaptive immunity, immune cells, melanocyte damage, CD8+ T cells

## Abstract

Vitiligo is an autoimmune disease characterized by the progressive destruction of epidermal melanocytes, leading to skin depigmentation. Although significant advances have been made in understanding the pathogenesis of vitiligo, the intricate interplay between genetic predisposition, environmental factors, oxidative stress, and immune dysregulation remains inadequately understood. In particular, increasing evidence highlights the pivotal role of innate immune activation in initiating and amplifying the adaptive immune response, particularly the activation of autoreactive CD8^+^ T cells, which are the ultimate effectors of melanocyte destruction. However, current therapeutic approaches offer limited efficacy in modulating this pathway. This review provides a comprehensive analysis of the mechanisms driving innate immune activation in vitiligo, with a particular focus on damage-associated molecular patterns (DAMPs), oxidative stress, and key innate immune cells, including dendritic cells (DCs), natural killer (NK) cells, and innate lymphoid cells (ILCs), and their crucial role in bridging innate and adaptive immunity. We further explore how these factors initiate and sustain an inflammatory cascade that bridges innate stress responses with adaptive immune activation, ultimately exacerbating melanocyte destruction. By synthesizing recent advances, we aim to elucidate the critical role of innate immunity in shaping disease progression and discuss emerging innate immune-targeted therapeutic strategies. Understanding these pathways may open new avenues for more effective and targeted interventions in vitiligo treatment.

## 1 Introduction

Vitiligo is an autoimmune skin disease characterized by the destruction of epidermal melanocytes, resulting in the loss of pigmentation in the skin, hair, and other tissues (1). Approximately, the prevalence of vitiligo is 0.5%-2% of the total population, and the number of Chinese vitiligo patients was about 20 million in 2021 (2, 3). Although vitiligo is not life-threatening, its visible manifestation often leads to significant psychological distress. The pathogenesis of vitiligo is complex, involving genetic, environmental, and immune factors, including theories of autoimmunity and oxidative stress (4). However, no single theory fully explains its etiology. The progressive loss of melanocytes is driven by a dysregulated immune response in which autoreacttive CD8^+^ T cells play a critical role. For instance, these cells identify melanocytes via Major Histocompatibility Complex (MHC) class I molecules and induce apoptosis through the directed release of perforin and granzyme B, while IFN-γ secretion exacerbates local inflammation and amplifies the autoimmune response, constituting a key pathological event in vitiligo (5).

**Figure 1:**
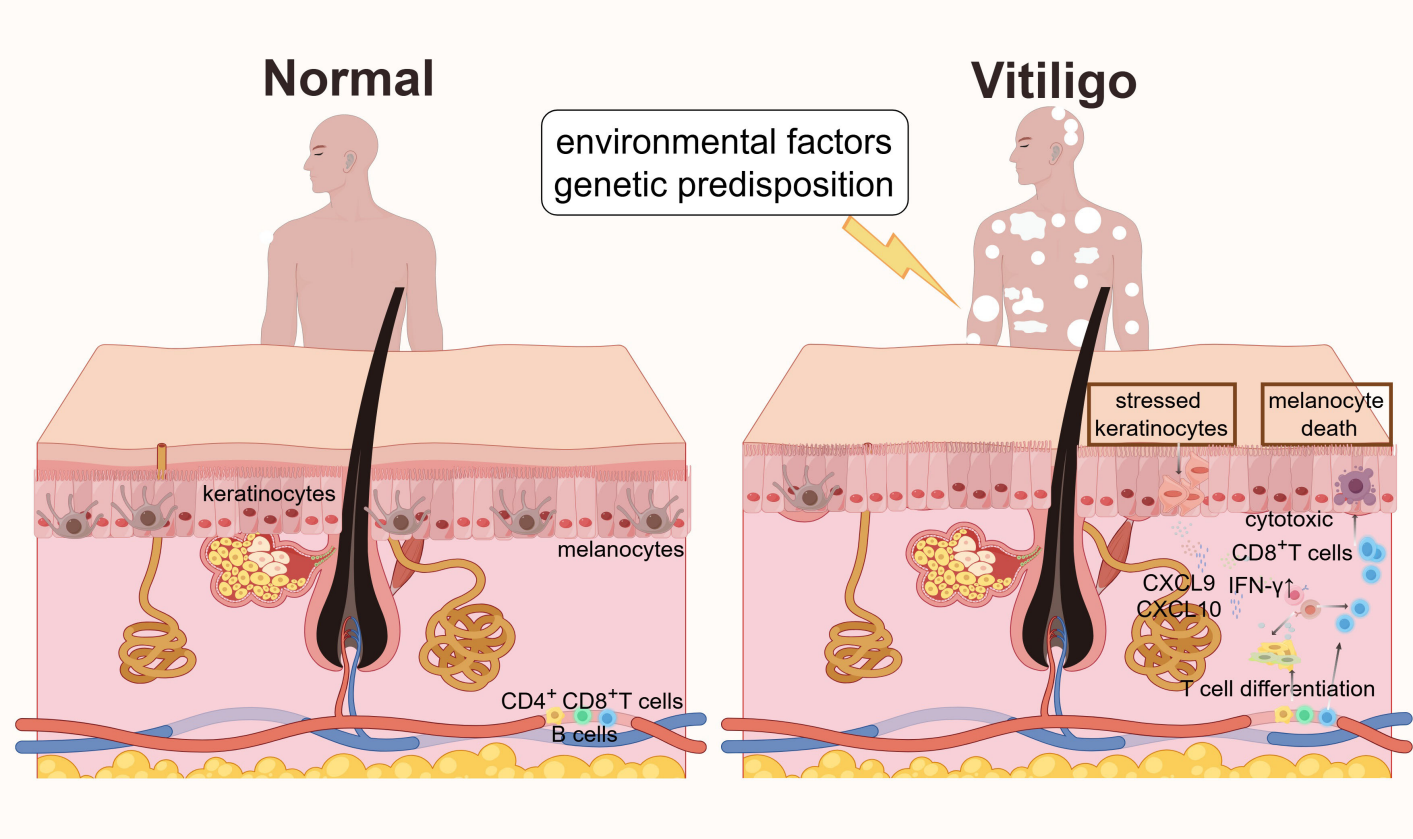
Mechanisms of vitiligo pathogenesis. In normal skin, melanocytes are evenly distributed in the basal layer of the epidermis. In vitiligo, environmental factors (such as oxidative stress) and genetic predisposition induce the release of DAMPs, activating PRRs in melanocytes and keratinocytes, accompanying the secretion of IFN-γ. Stressed keratinocytes release cytokines and chemokines (such as CXCL9 and CXCL10) to recruit cytotoxic CD8^+^ T cells. These cascades lead to melanocyte death, disrupted pigmentation, and the characteristic depigmented skin lesions of vitiligo.

Recent research highlights that environmental factors, such as ultraviolet (UV) radiation or chemical exposure, can trigger vitiligo by inducing the production of pathogen-associated molecular patterns (PAMPs) or damage-associated molecular patterns (DAMPs) (6). These molecules interact with NLRP1 in the cytoplasm of dendritic cells (DCs), initiating the innate immune response. This process subsequently leads to adaptive immune activation, where CD8^+^ T cells target melanocytes, resulting in skin depigmentation. Thus, the activation of the innate immune system plays a key role in the onset and progression of vitiligo, largely by creating a pro-inflammatory microenvironment and orchestrating the activation and recruitment of pathogenic CD8^+^ T cells. This article summarizes the various stressors of innate immune activation and their regulation of adaptive immunity, with a specific emphasis on how innate immune mechanisms converge to activate CD8^+^ T cells, as well as the role of these processes in the pathological mechanism of vitiligo (**Figure 1**).

## 2 The role of innate immunity in vitiligo

The innate immunity system serves as the body’s first line of defense, capable of recognizing and responding to pathogens without prior exposure (7). This system includes physical barriers (such as skin), immune cells (such as macrophages, neutrophils, and DCs), and a range of cytokines and complement systems (8, 9). In vitiligo specifically, abnormal activation of innate immunity is considered a crucial factor driving disease progression. Elevated expression of innate immune-related genes has been reported in both lesional and non-lesional skin of vitiligo patients (10–12). Notably, oxidative stress has been shown to activate immune cells such as DCs and NK cells, which are pivotal in the early onset of vitiligo (13, 14). Critically, the innate immune response sets the stage for the adaptive arm by promoting the activation, antigen presentation, and migration of CD8^+^ T cells, the principal executors of melanocyte destruction (15).

## 3 Factors triggering innate immune activation in vitiligo

**Figure 2:**
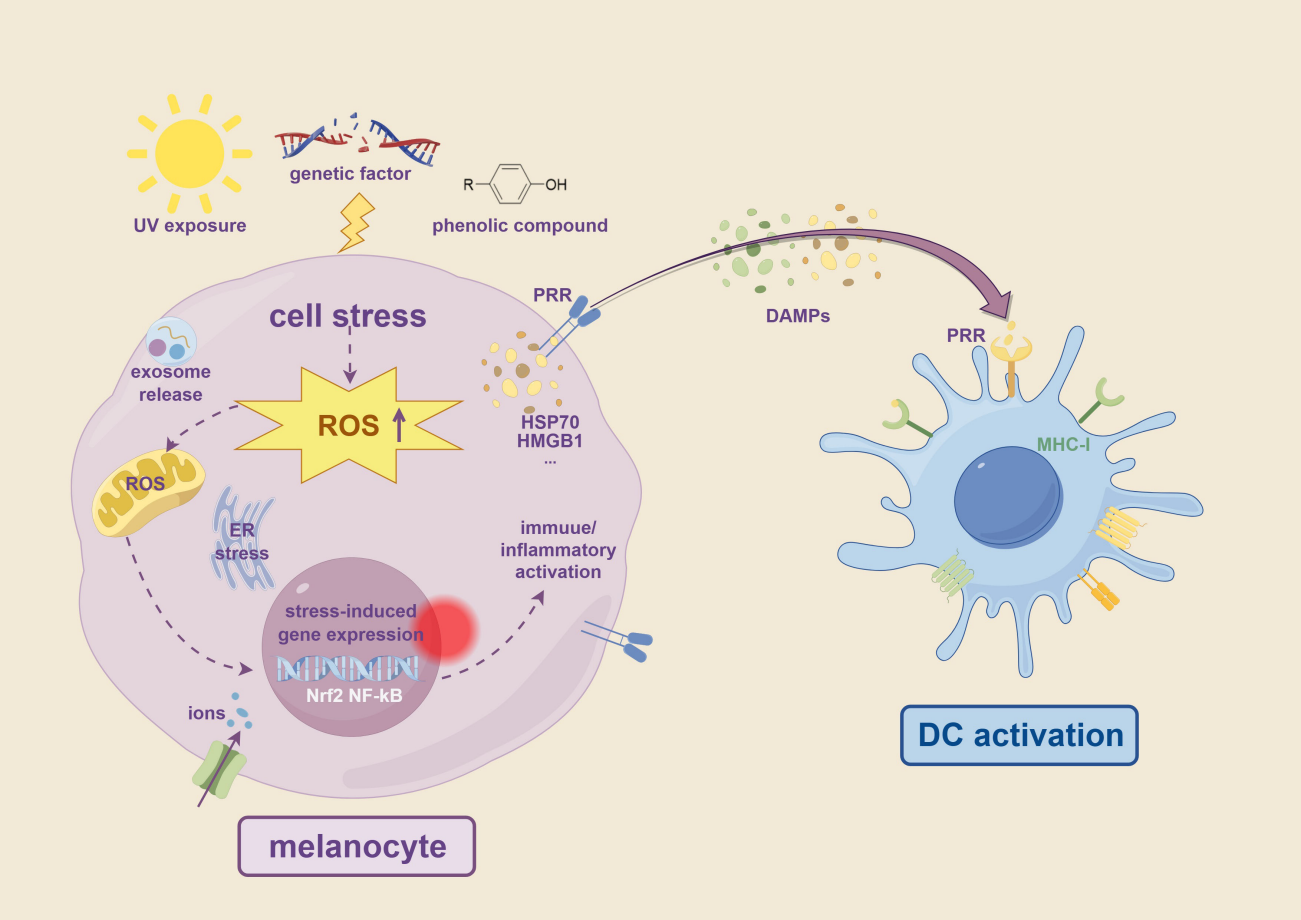
Overview of factors triggering innate immune activation in vitiligo. Environmental stressors such as UV radiation, phenolic compounds, and genetic predisposition lead to cell stress in melanocytes, characterized by increased ROS production. This oxidative stress induces ER stress, ion imbalance, including iron and copper ions overload, and activation of stress-responsive transcription factors such as Nrf2 and NF-κB. These cellular stress responses promote the release of DAMPs, including HSP70, HMGB1, and exosomes. DAMPs are recognized by PRRs such as TLRs and NLRs on DCs, leading to immune activation and antigen presentation through MHC-I. These processes initiate innate immune responses that contribute to melanocyte destruction in vitiligo.

Innate immune activation in vitiligo is triggered by a variety of factors, including environmental, genetic, and endogenous elements. These factors induce oxidative stress, endoplasmic reticulum (ER) stress, or DAMPs release, resulting in inflammation, immune dysregulation, and melanocyte destruction (16, 17). For example, UV exposure has been shown to directly damage melanocytes (18). Simultaneously, certain chemicals, such as phenolic compounds, can either directly destroy melanocytes or induce an immune response by triggering oxidative stress (19). Genetic and epigenetic factors also influence the innate immune system. Variants in certain genes, such as *NLRP1* and *IFIH*, may predispose individuals to activate innate immune pathways more readily. The environment can also alter DNA methylation or histone modifications, regulating the expression of inflammatory genes and enhancing the innate immune response (20). These extrinsic factors and intracellular stress collectively drive a series of emergency mechanisms, forming a complex network of immune dysregulation (**Figure 2**). This network not only directly stresses melanocytes but also potently modulates the adaptive immune system, particularly by priming and recruiting CD8^+^ T cells. Understanding these driving mechanisms will be instrumental in elucidating the pathophysiological mechanisms of vitiligo.

### 3.1 Reactive oxygen species and oxidative stress

Oxidative stress is widely recognized as a primary trigger of vitiligo. Melanocytes generate large amounts of ROS during melanin synthesis, which under normal conditions are neutralized by antioxidant systems such as glutathione and superoxide dismutase (SOD) (21, 22). However, in vitiligo patients, diminished antioxidant capacity leads to ROS accumulation, inducing cellular damage and apoptosis (23). High levels of hydrogen peroxide (H_2_O_2_) have been detected in the epidermis of vitiligo patients (24). Yuan et al. confirmed that H_2_O_2_ induced apoptosis in PIG1 and PIG3V cells, but treatment with antioxidants could alleviate melanocyte damage (25). Past studies have suggested that oxidative stress may be the initial event of melanocyte damage (26, 27). Moreover, oxidative stress also activates pattern recognition receptors (PRRs), such as Toll-like receptors (TLRs) and NLRP1, further amplifying the innate immune response and triggering chronic inflammation (9). Furthermore, oxidative stress enhances the migration of CD8^+^ T cells to the skin, thereby linking innate immune activation to adaptive immunity. Li et al. demonstrated that oxidative stress drives CD8^+^ T cells’ skin trafficking in patients with vitiligo through CXCL16 upregulation in keratinocytes (28). Importantly, ROS-induced chemokine release (such as CXCL16) provides a direct link to CD8^+^ T cells recruitment, underscoring the convergence of oxidative stress with adaptive immunity.

### 3.2 Heat shock protein 70 and stress response

Heat shock proteins (HSPs) are molecular chaperones that help maintain cellular homeostasis under stress conditions (29). HSP70, particularly its inducible form (HSP70i), is upregulated in vitiligo and serves as a “danger signal” that activates the innate immune response (30). In the early stages of vitiligo, oxidative stress may induce HSP70 expression to protect melanocytes. However, excessive oxidative stress leads to the extracellular release of HSP70, which triggers immunity cascades. Jacquemin et al. reported that HSP70 potentiates interferon alpha (IFN-α) production by plasmacytoid DCs, which further highlights that HSP70 acts as a danger signal for vitiligo (31). Moreover, exogenous HSP70 activates DCs and NK cells by binding to TLR2 and TLR4. This reaction triggers a local inflammatory response, promotes the infiltration of immune cells, and activates adaptive immunity, ultimately triggering the destruction of melanocytes (32). Furthermore, Mosenson et al. demonstrated that altering the substrate-binding domain of HSP70i by using HSP70iQ435A-encoding DNA reduced DC activation and skin T cell infiltration. This approach prevented white spot formation in a mouse model of vitiligo and promoted the recovery of pigmentation (33). These findings indicate that HSP70 is both a protector of cell function and a promoter of immune response. The activation of DCs and NK cells by HSP70 ultimately promotes CD8^+^ T cells priming and cytotoxicity, reinforcing the central role of these lymphocytes in melanocyte destruction.

### 3.3 Endoplasmic reticulum stress and immune activation

ER stress occurs when misfolded or unfolded proteins accumulate beyond the ER’s processing capacity, activating the unfolded protein response (UPR) (34). Tyrosinase is a key enzyme for melanin synthesis, and its correct folding and function are carried out in the ER (35). When folding stress occurs in the ER, the misfolding of tyrosinase affects its function, thereby inhibiting melanogenesis. Oyama’s study demonstrated that hinokitiol-induced reduction of tyrosinase in human melanoma cells was mediated by ER-related degradation pathways (36). Similarly, H_2_O_2_-induced ER expansion and swelling inhibit ER function and reduce tyrosinase production in melanocytes (37). Importantly, ER folding stress activates innate immune responses through multiple pathways. Initially, ER folding stress activates the UPR and promotes the secretion of inflammatory factors through IRE1, NF-κB, and JNK signaling pathways, thereby activating the innate immune response (38–40). Furthermore, misfolded proteins act as DAMPs to stimulate the activation of DCs and macrophages, promoting the migration of CD8^+^ T cells, leading to adaptive immunity activation. Song et al. reported that treatment with calreticulin in melanocytes can activate UPR, and calreticulin can promote the migration of CD8^+^ T cells in vitiligo (41). Intriguingly, the study reported that activating the UPR in keratinocytes can promote CXCL16 upregulation and drive CD8^+^ T cells’ skin transport in vitiligo patients. These findings indicate that ER stress can regulate melanogenesis and activate the innate immune response, aggravating the pathological process of vitiligo. Thus, ER stress not only compromises melanogenesis but also creates inflammatory signals that facilitate CD8^+^ T cell migration and activation, establishing a bridge to adaptive immunity.

### 3.4 Exosomes and miRNAs in immune regulation

Exosomes are extracellular vesicles that mediate intercellular communication by transferring proteins, lipids, and miRNAs (42). In melanocytes, the release of exosomes is activated under stress conditions, and these exosomes carry melanocyte-specific antigens such as tyrosinase (TYR), tyrosinase-related protein 1 (TYRP-1), and tyrosinase-related protein 2 (TYRP-2), which can be taken up by DCs to activate the immune response (43). Meanwhile, miRNAs in exosomes also play a key role in regulating the immune response. For example, miR-125b-5p, highly expressed in melanocytes, has been shown to inhibit the expression of TYR, TYRP-1, and TYRP-2, leading to melanocyte apoptosis. Meanwhile, miR-132-3p is involved in the regulation of Treg cell differentiation, which is essential for maintaining immune homeostasis and preventing autoimmunity (44). These studies not only enhance our comprehension of the roles exosomes and miRNAs play in melanocyte biology but also identify potential targets for devising innovative therapeutic strategies. Notably, the transfer of melanocyte antigens via exosomes to DCs represents a crucial link between stressed melanocytes and the initiation of CD8^+^ T cell responses, facilitating the breakdown of immune tolerance.

### 3.5 High mobility group box 1 and inflammatory signaling

HMGB1, a nuclear protein, functions as a transcription factor and a DAMP that amplifies inflammatory responses (45). Clinical studies have demonstrated that HMGB1 was overexpressed in the serum and skin of vitiligo patients compared to healthy controls (46). Kim et al. demonstrated that HMGB1 can affect the survival of melanocytes and regulate the expression of molecules related to melanin synthesis by binding to cell surface receptors (47). Meanwhile, the release of HMGB1 may promote the development of autoimmune responses and induce melanocyte death (48). Studies have reported that oxidative stress and UV exposure induce HMGB1 release from keratinocytes, which then binds to RAGE and TLRs, activating NF-κB and MAPK signaling, consequently promoting the production of inflammatory cytokines such as IL-1β, IL-6, and TNF-α (46, 49). Consequently, HMGB1 is not only involved in the regulation of melanocyte survival and function but also exacerbates vitiligo progression by activating inflammatory and autoimmune responses. The pro-inflammatory cytokines driven by HMGB1 signaling contribute to the microenvironment that supports CD8^+^ T cell activation and function.

### 3.6 Ferroptosis: iron-dependent cell death

Ferroptosis, a non-apoptotic form of regulated cell death involving iron ions and lipid peroxidation, is characterized primarily by the accumulation of lipid peroxides in the cell membrane (50). Abnormal iron metabolism in vitiligo patients may aggravate oxidative stress and induce ferroptosis in melanocytes through lipid peroxidation (51).

Recent studies have indicated that ferroptosis, as an important mechanism of melanocyte destruction, not only directly leads to cell death but also intensifies the inflammatory response and immune dysregulation in vitiligo by enhancing oxidative stress and activating innate immune pathways (52, 53). For instance, excessive iron promotes the Fenton reaction, generating more ROS, which further damages melanocytes (54). Excessive iron promotes the Fenton reaction, generating more ROS, further damaging melanocytes, while glutathione peroxidase 4 (GPX4), a key regulatory factor in ferroptosis, shows significantly downregulated expression in vitiligo. Studies have demonstrated that the expression of GPX4 was significantly downregulated in vitiligo and that exogenous supplementation of GPX4 or iron chelators can alleviate melanocyte damage (55). Furthermore, ferroptosis may also exacerbate inflammatory responses by enhancing the activation of the innate immune system. Lipid peroxidation products can be recognized by DCs as DAMPs, activating the TLR4 and NLRP3 inflammasome pathways, and intensifying local immune dysregulation (56). The innate immune response, activated by DAMPs, thereby primes the adaptive CD8^+^ T cell response against melanocytes. These mechanisms may provide an important direction for an in-depth study of the pathogenesis of vitiligo.

### 3.7 Cuproptosis: a novel cell death pathway

Cuproptosis is a newly discovered, unique form of cell death induced by the direct binding of copper ions to lipoylated proteins, disrupting the function of protein-associated iron-sulfur clusters and triggering cell death. Research has indicated that the concentration of copper in the serum of Iranian vitiligo patients was significantly lower compared to healthy individuals (57). Abnormal local concentrations of copper ions may induce melanocyte cuproptosis and exacerbate disease progression.

During cuproptosis, the aberrant intracellular accumulation of copper ions induces mitochondrial dysfunction, leading to excessive ROS production and amplifying oxidative stress (58). Furthermore, copper ions can disrupt protein structures, inducing ER stress and aggravating cell injury and death (59). Similar to ferroptosis, cuproptosis may also release DAMPs, which activate inflammatory pathways and trigger innate immune responses. Notably, it has been reported that the upregulation of HSP70 is closely linked to cuproptosis, and HSP70 is a crucial factor in activating innate immune response and aggravating the pathological changes in vitiligo (60, 61). The above reports indicated that there was an association between copper and vitiligo, highlighting the potential role of cuproptosis in vitiligo and warranting further attention. However, to date, there have been no definitive research reports directly linking cuproptosis to the pathogenesis of vitiligo, making it a promising area for future exploration. Cuproptosis may collaborate with CD8^+^ T cells by inducing innate DAMPs that enhance T cell recruitment. Independently, cuproptosis-driven innate mitochondrial stress could directly lead to melanocyte loss, offering a novel T cell-independent pathway for vitiligo progression.

## 4 Mechanisms of innate immune activation in vitiligo: a multi-faceted immune response

Building on the triggering factors discussed above, vitiligo is an autoimmune disorder characterized by the progressive destruction of melanocytes, leading to depigmented skin lesions. The innate immune system plays a crucial role in its pathogenesis by mediating early inflammatory responses and facilitating the activation of adaptive immunity, culminating in the execution of melanocyte destruction by CD8^+^ T cells. This section explores three key mechanisms involved in the innate immune activation in vitiligo: the release of DAMPs and PRRs activation, DCs’ antigen presentation, and the engagement of innate lymphoid cells (ILCs).

### 4.1 DAMPs release and PRRs activation

Upon encountering oxidative stress, chemical injury, or other extrinsic factors, melanocytes generate DAMPs, such as HSP70i and HMGB1 (62). These DAMPs are recognized by PRRs in the skin (such as TLRs, NLRs), which trigger downstream signaling pathways like NF-κB and MAPK pathways, and promote the production of inflammatory cytokines such as TNF-α, IL-8, and IL-1β (16, 63). Notably, the study has demonstrated an upregulation of TLR7 and TLR9 in melanocytes of vitiligo patients, suggesting a heightened state of innate immune activation in this condition (64). Moreover, a study has shown that HMGB1 can bind to the receptor for RAGE, activating the NF-κB signaling pathway, and consequently promoting the production of IL-8 and CXCL16 in keratinocytes.

These inflammatory cytokines not only amplify the inflammatory response but also facilitate the activation and recruitment of immune cells, such as DCs and NK cells, which play a pivotal role in the pathogenesis of vitiligo (65). In vitiligo, DAMPs enhance the maturation of DCs, elevating the expression levels of co-stimulatory molecules such as CD80 and CD86, thereby augmenting their antigen-presenting capabilities (66). Additionally, DAMPs stimulate the secretion of chemokines like CCL21 and CCL19 by DCs, which attract their migration toward lymph nodes, activate CD8^+^ T cells, and initiate specific immune responses (67). This process is fundamental for the priming of unprimed CD8^+^ T cells against melanocyte antigens.

In vitiligo, the release of DAMPs and the activation of PRRs are also closely related to the injury and apoptosis of melanocytes. DAMPs can trigger the production of inflammatory factors by activating PRRs, which can further damage melanocytes, leading to apoptosis and loss of function of melanocytes (68). Moreover, by promoting DC maturation and migration, DAMPs help trigger CD8^+^ T cells activation in the lymph nodes, further exacerbating melanocyte damage. Therefore, the release of DAMPs and the activation of PRRs bridge initial tissue damage to the pathogenesis of vitiligo, providing a new target for the treatment of vitiligo.

### 4.2 DCs antigen presentation dendritic cell-mediated antigen presentation: linking innate and adaptive immunity

DCs are key antigen-presenting cells (APCs) that serve as a bridge between innate and adaptive immunity (69). Studies have indicated that both the number and activity of DCs are significantly elevated in the skin and peripheral blood of vitiligo patients (70). These DCs are capable of capturing and processing antigens from melanocytes, such as Melanoma-Associated Antigen Recognized by T cells 1 (MART-1) and TYR, and presenting these antigens to T cells via MHC molecules (71, 72). The presentation of melanocyte-derived peptides on MHC class I molecules is the critical event that enables CD8^+^ T cells to recognize and target melanocytes. The expression of co-stimulatory molecules on DCs, including CD80 and CD86, is notably increased in vitiligo patients (73), enhancing their antigen-presenting ability and promoting the activation and proliferation of T cells, particularly CD8^+^ T cells, which contribute to the destruction of melanocytes.

For example, the abnormal activation and dysregulation of antigen-presenting functions of DCs are intricately linked to multiple factors. The release of DAMPs and subsequent activation of PRRs enhance the maturation and migration of DCs, which, in turn, activate T cells in lymph nodes. Inflammatory cytokines such as TNF-α, IL-6, and IL-1β further potentiate DC activation and antigen presentation (74). Moreover, innate immune mediators such as IFN-γ, secreted by natural killer (NK) cells and innate lymphoid cells, can upregulate HLA class I expression on melanocytes, thereby amplifying CD8^+^ T cell recognition (75). In parallel, TLR signaling within DCs promotes HLA molecule upregulation and drives their maturation, further strengthening the interface between innate and adaptive immunity. However, the precise mechanisms through which these cytokines regulate DC function in vitiligo remain poorly understood.

The dysfunction of antigen presentation by DCs is closely related to the damage and apoptosis of melanocytes (76). Studies have shown that DCs can activate specific T cells by presenting melanocyte antigens, leading to melanocyte damage and apoptosis (77). In addition, DCs can secrete cytokines such as TNF-α and IFN-γ, further harming melanocytes and resulting in their apoptosis and functional loss (78). Consequently, the abnormal activation and impaired antigen-presenting function of DCs, together with innate immune modulation of HLA expression, play a critical role in the pathogenesis of vitiligo, providing new therapeutic targets for the treatment of this condition. Targeting DC antigen presentation or their interaction with CD8^+^ T cells could therefore disrupt a key step in the autoimmune cascade.

### 4.3 The engagement of innate lymphoid cells: amplifying immune activation

ILCs are a diverse family of immune cells that act as critical mediators between innate and adaptive immunity. They are classified into several subtypes, including NK cells, ILC1, ILC2, ILC3, and lymphoid tissue inducer (LTi) cells (79). Among them, NK cells play a particularly significant role in vitiligo by exerting direct cytotoxic effects on melanocytes and shaping the broader immune response (80–82). Furthermore, ILCs can profoundly influence the activity of CD8^+^ T cells through cytokine secretion.

#### 4.3.1 NK cells: key players in melanocyte destruction

NK cells contribute to melanocyte destruction through three main mechanisms. First, they exhibit potent direct cytotoxicity, lysing melanocytes via the release of perforin and granzyme B. Second, NK cells target upregulated NKG2D ligands on melanocytes (such as MHC class I-related protein A), triggering cytotoxic responses (83). Third, NK cells activate other immune cells, such as T cells, through cytokine secretion and chemokine-mediated migration, thereby priming adaptive immunity. In particular, NK cell-derived IFN-γ is a potent activator of macrophages and DCs and promotes CD8^+^ T cell differentiation and cytotoxic function, forming a pro-inflammatory loop. Studies confirm a significant increase in NK cell numbers in the peripheral blood of vitiligo patients (84). CD16^+^ and CD56^+^ cells (markers of NK cell activity) are notably elevated in peripheral blood (85, 86). Early studies reported that NK cells exacerbate vitiligo progression by directly killing melanocytes (87). More importantly, NK cells further damage melanocytes by secreting IFN-γ, recruiting adaptive immune responses, and aggravating melanocyte destruction. Recent reports indicate that NK cells attract other immune cells, such as T cells and DCs, to inflammatory sites by secreting chemokines. Tulic et al. demonstrated that NK cells upregulate the expression of chemokines like CXCL9 and CXCL10 via IFN-γ secretion (88). These chemokines induce melanocyte apoptosis by activating the CXCR3B receptor. Simultaneously, melanocytes express co-stimulatory molecules (e.g., CD40, CD80) and adhesion molecules (e.g., ICAM-1), enabling them to present self-antigens to T cells, thereby promoting T cell proliferation and activation, ultimately contributing to vitiligo progression. The recruitment of CXCR3^+^ CD8^+^ T cells to the skin via NK cell-derived CXCL9/CXCL10 is a critical mechanism linking innate NK cell activity with adaptive T cell-mediated destruction.

#### 4.3.2 Other innate lymphoid cells in vitiligo

Besides NK cells, other ILCs, such as ILC1, ILC2, and ILC3, contribute to immune surveillance and regulation, further emphasizing their importance in autoimmune diseases like vitiligo. Similar to NK cells, ILC1s can secrete IFN-γ and TNF-α, triggering local inflammation and enhancing adaptive immune responses (89). Additionally, ILC1 promotes a sustained immune activation state by secreting CXCL9 and CXCL10, recruiting DCs and T cells to inflammatory sites (90). However, unlike NK cells, ILC1s lack direct cytotoxic capacity and do not contain perforin or granzyme B. Compared to ILC1 and NK cells, ILC2 has been less studied in vitiligo. Studies suggest that ILC2 is upregulated in allergic diseases (e.g., atopic dermatitis) and may play a role in vitiligo. On the other hand, ILC3 is upregulated in psoriatic skin, producing mediators like IL-17 and IL-22, which contribute to mucosal immunity and tissue repair (91). The potential role of ILC1-derived IFN-γ in supporting a Type 1 microenvironment conducive to CD8^+^ T cell function warrants further investigation in vitiligo.

## 5 Regulation of adaptive immunity by innate immunity in vitiligo

**Figure 3:**
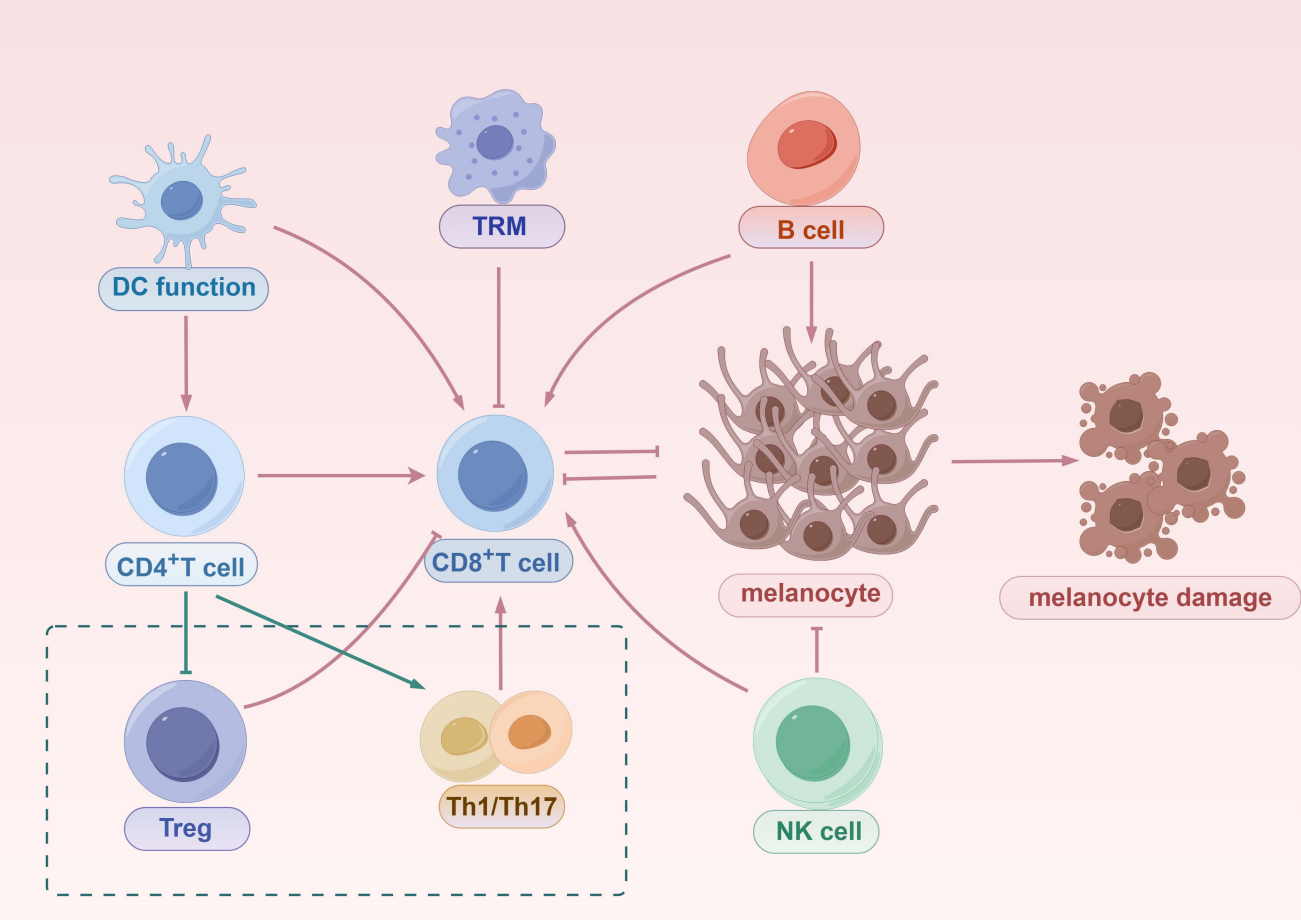
The mechanisms of innate and adaptive immune activation in vitiligo. Innate immune system, initiated by DCs, promotes the priming and activation of CD8^+^ T cells, which migrate to the skin and directly mediate melanocyte destruction. DCs also activate CD4^+^ T cells, which can differentiate into Tregs that suppress CD8^+^ T cell responses or into Th1/Th17 effector subsets that contribute to inflammation and further recruitment of cytotoxic T cells. TRMs, derived from CD8^+^ T cells, persist in the skin and contribute to disease recurrence. B cells amplify autoimmunity through autoantibody production and by enhancing CD8^+^ T cell responses. NK cells function both as innate effectors capable of directly killing melanocytes and as modulators that enhance CD8^+^ T cell activity. Together, these innate and adaptive components form an interconnected immune network that drives progressive melanocyte loss in vitiligo.

Vitiligo is an autoimmune disease driven by dysregulated adaptive immunity, where autoreactive CD8^+^ T cells selectively destroy melanocytes, leading to progressive skin depigmentation. The interplay between innate and adaptive immunity is complex and critical in vitiligo’s pathogenesis. The innate immune system not only serves as the first line of defense but also shapes adaptive immune responses through cytokine and chemokine secretion, co-stimulatory signals, and direct immune cell interactions (**Figure 3**). This section elaborates on the mechanisms by which innate immunity regulates adaptive immunity in vitiligo, emphasizing the interconnected processes driving disease progression, with a focus on CD8^+^ T cell activation and function.

### 5.1 Cytokines and chemokines secretion

Cytokines and chemokines are pivotal mediators linking innate and adaptive immune responses (92). These molecules are essential for amplifying inflammation and recruiting immune cells while directly influencing T cell activation, differentiation, and function (93). In vitiligo patients’ skin, activated innate immune cells (e.g., DCs and NK cells) secrete multiple pro-inflammatory cytokines and chemokines, including IL-1β, IL-6, IFN-γ, TNF-α, CXCL10, and CCL2 (94, 95). These cytokines establish a highly inflammatory microenvironment in the skin, attracting T cells and other immune cells to affected areas, thereby promoting localized inflammation progression. Ben-Sasson et al. reported that IL-1β significantly enhances the expansion of antigen-primed CD4^+^ and CD8^+^ T cells *in vivo* (96). This indicates that innate cytokine signaling directly amplifies the CD8^+^ T cell pool.

### 5.2 Chemokines: bridging innate and adaptive immunity

Notably, IFN-γ-induced chemokine expression, such as CXCL9 and CXCL10, is significantly elevated in the lesional skin and plasma of vitiligo patients, often serving as biomarkers of clinical activity (97, 98). Infiltrating CD8^+^ T cells exacerbate melanocyte damage by secreting additional IFN-γ and granzyme B (99). Additionally, CCL2 promotes the recruitment of monocytes and macrophages to the skin, further amplifying the inflammatory response (100, 101).

Chemokines not only play a critical role in recruiting innate immune cells but also serve as a vital bridge in initiating and sustaining adaptive immunity. By regulating immune cell migration and localization, chemokines determine the magnitude and strength of specific immune responses. The interaction between CXCL10 and its receptor CXCR3 is particularly significant in autoimmune diseases. As mentioned, CXCL10, significantly elevated under IFN-γ induction, guides CD8^+^ T cells to lesion sites via CXCR3. Conversely, studies have shown that CXCL10-neutralizing antibody treatment blocks CD8^+^ T cell skin recruitment, significantly inhibiting disease progression in vitiligo mouse models (102). CXCL16, derived from keratinocytes under oxidative stress, could also recruit CD8^+^ T cells to the epidermis, resulting in melanocyte damage in vitiligo (103). This precise regulation of chemotactic response prolongs the intensity and duration of local immune reactions, thereby exacerbating melanocyte destruction (104).

Moreover, CCL5 (known as RANTES) plays a significant role in the establishment and maintenance of the inflammatory microenvironment in vitiligo by chemotactically attracting monocytes and macrophages via its receptor CCR5 (105, 106). By regulating chemokine expression, the innate immune system effectively coordinates adaptive immune responses, ensuring various immune cell types function within the appropriate spatial and temporal framework. The spatial guidance provided by innate-derived chemokines is critical for directing CD8^+^ T cells to target melanocytes within the epidermis. Chemokine-receptor interactions also establish a positive feedback loop between innate and adaptive immunity. For instance, elevated CXCL10 in vitiligo lesions not only attracts CD8^+^ T cells but also stimulates these cells to secrete additional IFN-γ, which further enhances CXCL10 expression, forming a self-amplifying inflammatory cycle. This feedback mechanism impedes spontaneous resolution of inflammation, contributing to chronic disease progression and recurrence. Disrupting the IFN-γ-CXCL9/10-CXCR3 axis represents a promising therapeutic strategy to impair CD8^+^ T cell recruitment.

### 5.3 T cell activation by co-stimulatory signals

In addition to cytokine and chemokine-mediated regulation, the innate immune system provides essential co-stimulatory signals for T cell activation. These signals are primarily delivered by DCs and macrophages, which not only capture and present antigens but also express co-stimulatory molecules and MHC molecules to deliver the second signal to T cells. This second signal is critical for T cell activation; without it, T cells encountering antigens may fail to activate fully or enter an anergic state. For CD8^+^ T cells, antigen peptide recognition on MHC class I molecules by professional APCs (such as DCs) provides signal one, while CD80/CD86-CD28 interactions provide the critical co-stimulatory signal two (107). In vitiligo, activated DCs migrate to regional lymph nodes, where they upregulate co-stimulatory molecules like CD80 and CD86, present melanocyte antigens to T cells, and provide the second signal for full activation. Additionally, DC-derived cytokines (such as IL-12 and IL-23) further regulate T cell differentiation, promoting the development of Th1 and Th17 immune responses (108).

Macrophages also contribute to vitiligo’s immune response by phagocytosing apoptotic melanocytes, uptaking melanocyte antigens, expressing co-stimulatory molecules, and activating T cells (109). Furthermore, macrophage polarization states (M1 or M2) play a critical role in regulating the type of local immune response. M1 macrophages primarily secrete pro-inflammatory cytokines, such as IL-12 and TNF-α, promoting Th1-type immune responses. However, M2 macrophages tend to secrete anti-inflammatory factors, such as IL-10, potentially aiding inflammation resolution in later stages (110, 111). The M1 macrophage phenotype, induced by innate signals such as IFN-γ, supports cytotoxic CD8^+^ T cell activation and function.

### 5.4 CD8^+^ T cells as central effectors in vitiligo pathogenesis

CD8^+^ cytotoxic T lymphocytes are central to vitiligo’s autoimmune pathogenesis, serving as the primary immune effector cells causing melanocyte damage and pigment loss (112). CD8^+^ T cells recognize specific antigens on the surface of melanocytes and directly induce cell death via cytotoxic mechanisms, leading to skin depigmentation (113). As previously noted, innate immune cells like DCs prime these CD8^+^ T cells by presenting melanocyte antigens via MHC class I. DC-derived cytokines (such as IL-12, IL-15) and chemokines (such as CXCL9, CXCL10) promote CD8^+^ T cell activation, proliferation, and directed migration.

The primary effector mechanism of CD8^+^ T cells is direct cytotoxicity. Upon recognizing and binding specific antigens (e.g., TRP1, TRP2) on melanocytes via their T cell receptors, CD8^+^ T cells initiate cytotoxic responses. These cells execute cytotoxicity by secreting perforin and granzyme B and releasing cytokines such as IFN-γ and TNF-α (114). Perforin and granzyme B work synergistically to form pores in the target cell membrane. After perforin forms pores in the cell membrane, granzyme B and other proteases enter the target cell and initiate programmed cell death (115). Additionally, IFN-γ and TNF-α can enhance the immune response of melanocytes by increasing their antigen-presenting capacity. Meanwhile, these cytokines may also contribute to melanocyte death in vitiligo. Notably, IFN-γ not only facilitates immune system recognition of melanocytes but also inhibits melanocyte proliferation and function, further exacerbating their damage.

Early studies reported activated CD8^+^ T cell infiltration at the dermal-epidermal junction in vitiligo lesions (116, 117). Clinical studies show that vitiligo lesions are often accompanied by localized immune cell infiltration, including CD8^+^ T cells, DCs, and NK cells (118). Immunohistochemical analysis of skin tissue reveals a significant increase in CD8^+^ T cell numbers in affected areas, with little to no presence in normal skin (119). Therapeutic approaches targeting CD8+ T cell activity, such as anti-CD8^+^ antibodies, have emerged as significant strategies for vitiligo treatment. Understanding the immunology of CD8^+^ T cell involvement in vitiligo opens doors to potential targeted interventions. Identifying the precise innate triggers leading to the breakdown of tolerance to melanocyte antigens presented by MHC class I is a critical frontier in vitiligo research.

### 5.5 T cell differentiation and immune dysregulation

Beyond cytotoxic CD8^+^ T cells, other T cell subsets, including CD4^+^ T helper (Th) cells, T regulatory (Treg) cells, and tissue-resident memory T (TRM) cells, are broadly involved in vitiligo’s pathogenesis (120, 121). The innate immune system not only induces the secretion of chemokines to affect the migration of T cells but also directly interacts with T cells to regulate their differentiation. In the immunopathological process of vitiligo, DCs and NK cells drive the differentiation of T cells into various effector subsets, such as Th1, Th17, and Treg cells, through multiple mechanisms (122).

DCs promote T cell differentiation into Th1 cells by secreting IL-12, which plays a critical role in IFN-γ production (123, 124). As noted, IFN-γ amplifies melanocyte destruction by activating CD8^+^ T cells. NK cells also promote Th1-type responses by secreting IFN-γ, indirectly influencing other innate immune cell functions (125). Elevated levels of IFN-γ in vitiligo lesions suggest that the Th1 immune response plays a dominant role in disease progression. Additionally, IL-23 is a key cytokine for Th17 cell differentiation, playing a significant role in various autoimmune diseases. IL-17, induced by Th17 cells, not only amplifies inflammation but also stimulates keratinocytes to produce chemokines, attracting numerous immune cells to lesion sites. The Th1-polarized environment, strongly influenced by innate immune cells, is critical for sustaining CD8^+^ T cell effector functions.

The innate immune system also influences immune imbalance in vitiligo by regulating Treg cell function and numbers. Treg cells negatively regulate CD8^+^ T cells. Studies show reduced Treg cell numbers in the peripheral blood and lesional tissues of vitiligo patients, along with decreased expression of Treg migration-related CCL22 (126, 127). Additionally, the expression of genes related to the immunosuppressive functions of Treg cells (such as IL-10, CTLA-4, and TGF-β) was downregulated, which weakened their ability to inhibit CD8^+^ T cell activity. Although IL-1 can be secreted by macrophages to promote Treg expansion, this regulatory mechanism is often disrupted in the inflammatory microenvironment of vitiligo, reducing Treg-mediated suppression of pathogenic T cells. The decrease in both the number and function of Treg cells promotes the excessive activation of CD8^+^ T cells, which perpetuates the autoimmune response in vitiligo. Innate immune signals may contribute to Treg dysfunction, thus unleashing CD8^+^ T cell activity.

Notably, TRM cells are also present in vitiligo lesions. These cells exhibit “immune memory” upon re-exposure to the same antigen, redifferentiating into effector T cells that secrete IFN-γ and induce melanocyte destruction. These mechanisms may underlie lesion recurrence post-treatment in vitiligo. Innate immune stimulation may contribute to the formation and/or maintenance of the TRM pool, ensuring rapid recall of CD8^+^ T cell responses upon challenge.

### 5.6 B Lymphocytes and humoral immunity in vitiligo

B lymphocytes are primarily responsible for humoral immune responses, recognizing foreign pathogens and producing targeted antibodies, forming a critical component of the adaptive immune system (128). Importantly, B lymphocytes are closely associated with the pathogenesis of various autoimmune diseases, including vitiligo. First, activated B cells differentiate into plasma cells, secreting antibodies like IgG and IgM to neutralize pathogens or mark antigens (129). These antibodies may target melanocyte-related antigens, such as TYR and TRP1, attacking melanocytes and leading to their depletion and white patch formation in the skin. Second, similar to DC antigen presentation, B cells bind antigens via B cell receptors (BCRs), process them into short peptide fragments via endocytosis, and present them with MHC class II molecules to Th1 and Th17 cells to activate T cells (130). Third, B cells secrete various cytokines, including IL-6 and IL-10, further activating T lymphocytes and forming a complex immune network (131). Finally, some B cells differentiate into memory B cells, enabling rapid responses upon re-exposure to the same antigen (132). Notably, B cell numbers are increased in the epidermis of vitiligo patients, and numerous melanocyte-specific antibodies related to disease activity are detected in patient sera. Clinical studies suggest that B cell depletion therapies (such as anti-CD20 antibody rituximab) are effective in certain autoimmune diseases (such as systemic lupus erythematosus) (133). However, their application in vitiligo requires further exploration. While the role of B cells and antibodies is recognized, their relative contribution to melanocyte destruction compared to CD8^+^ T cell-mediated cytotoxicity needs further clarification. Antibodies may promote melanocyte damage via Fc receptor-mediated mechanisms or complement activation, but CD8^+^ T cell responses remain the primary effector pathway.

## 6 Future perspectives

Recent advances in vitiligo management have introduced diverse therapeutic strategies targeting immune dysregulation, oxidative stress, and melanocyte regeneration. Traditional pharmacological therapies, including corticosteroids and calcineurin inhibitors, suppress aberrant immune activity and reduce inflammation, while antioxidants like vitamins C and E mitigate ROS accumulation (134). However, their efficacy is limited. Narrow-band UVB phototherapy remains a cornerstone for pigment regeneration, but its effectiveness varies significantly among individuals (135). Emerging nanotechnology-based drug delivery systems show transformative potential by enhancing transdermal absorption, improving drug targeting, and reducing off-target effects. For example, nano-encapsulated antioxidants (e.g., vitamin C, resveratrol) demonstrate dual benefits in scavenging ROS and stimulating melanogenesis (136).

Immune-modulating therapies represent a paradigm shift in vitiligo treatment, moving beyond symptomatic relief toward pathogenesis-directed interventions. The IFN-γ-CXCL9/10-CXCR3 axis, central to CD8^+^ T cell recruitment and melanocyte destruction, has emerged as a key target (137). In 2022, the U.S. FDA approved ruxolitinib cream as the first topical JAK inhibitor for vitiligo, demonstrating significant repigmentation in facial and body lesions, particularly in patients with early-stage disease (138, 139). Clinically, this approval has broadened treatment options for non-segmental vitiligo, with real-world data suggesting improved quality of life through reduced visible depigmentation and enhanced patient adherence due to its topical nature. Similarly, IFN-γ neutralizing antibodies have shown promise in preclinical models by inhibiting inflammatory cascades and protecting melanocytes from immune-mediated apoptosis (140). However, challenges such as incomplete repigmentation in acral areas or recurrence upon discontinuation highlight the need for combination therapies. We hypothesize that integrating these agents with innate immune modulators could enhance durability, as innate pathways may perpetuate low-level inflammation even after adaptive immunity is suppressed.

Beyond pharmacological innovations, advanced delivery technologies are redefining localized, patient-friendly therapies. Microneedles, as a minimally invasive platform, enable precise delivery of immunomodulators, antioxidants, or cell suspensions to the dermal-epidermal junction, bypassing the stratum corneum barrier. Studies have demonstrated that microneedle-mediated administration of tacrolimus, tofacitinib, or α-MSH achieves superior repigmentation compared to conventional topical or systemic routes, while reducing systemic exposure and associated risks like immunosuppression (141). Nanotechnology carriers, such as antioxidant-loaded liposomes or polymeric nanoparticles, further improve bioavailability and provide synergistic effects in alleviating oxidative stress and bolstering melanocyte survival (142). Clinically, these approaches could translate to reduced treatment burden, with microneedle patches offering at-home application and potentially higher compliance rates among pediatric or needle-averse patients. The relationship between innate and adaptive immunity underscores their value: innate triggers, such as DAMPs from stressed melanocytes, may initiate adaptive responses via antigen presentation, creating a self-amplifying loop. Targeting this crosstalk through combined delivery systems could disrupt both arms of the immune response more effectively.

Despite these advances, several mechanistic uncertainties remain. Current research predominantly emphasizes adaptive immunity, while the contribution of innate immune pathways remains incompletely understood (143). A critical unresolved question is how specific innate immune triggers disrupt tolerance to melanocyte antigens presented by MHC class I molecules. Furthermore, the relative roles of innate cytotoxic mechanisms versus the orchestration of adaptive CD8^+^ T cell responses across different disease stages remain unclear. In addition, innate-driven cell death modalities such as ferroptosis or cuproptosis may sustain depigmentation in the absence of robust T cell activity, potentially accounting for treatment resistance or disease relapse.

From our perspective, incorporating innate immunity into the current therapeutic paradigm may explain the heterogeneity of clinical outcomes. We hypothesize that innate amplification loops, operating independently or synergistically with adaptive responses, contribute to disease persistence. Addressing these mechanisms requires advanced methodologies, including single-cell multi-omics and spatial transcriptomics, to dissect the interplay between innate and adaptive compartments at lesion sites. Clinically, such insights may pave the way for combination strategies, for example pairing JAK inhibitors with modulators of innate immune activation, thereby improving long-term repigmentation stability.

In summary, future therapeutic innovations should move beyond simple immune suppression to a more integrated model that addresses oxidative stress, adaptive immunity, and innate immune amplification simultaneously. By aligning mechanistic insights with innovative delivery technologies, the next generation of vitiligo therapies could provide more personalized, durable, and clinically meaningful outcomes for patients.

## 7 Conclusion

**Figure 4:**
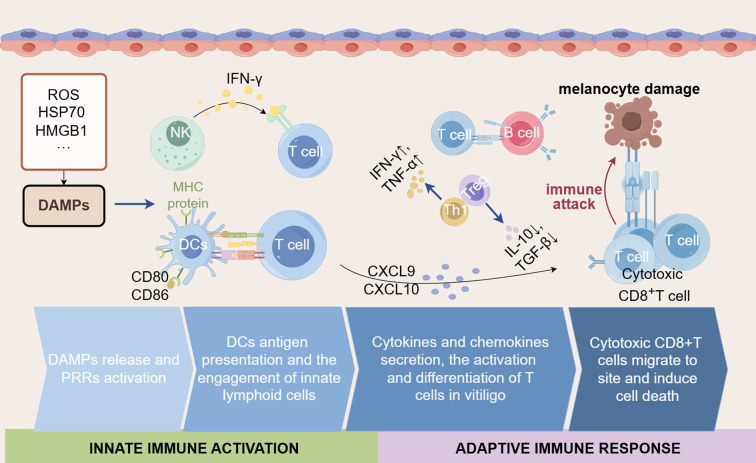
The interplay of innate immune activation and adaptive immune responses in vitiligo. The innate immune system plays a critical role in vitiligo by modulating the intensity and nature of adaptive immune responses through the secretion of various cytokines and chemokines. Initially, DAMPs (such as ROS, HSP70, and HMGB1) are released by stressed cells and initiate the innate immune response. During this process, the activated DCs present antigens via MHC proteins to T cells and express co-stimulatory molecules (such as CD80 and CD86), together with the engagement of NK cells, further amplifying the activation of T cells. Subsequently, cytokines (such as IFN-γ, TNF-α) and chemokines (such as CXCL9 and CXCL10) are secreted, which can recruit and activate CD8^+^ T cells. In vitiligo, despite attempts to regulate the immune response by Tregs and regulatory factors (such as IL-10 and TGF-β), the destruction of melanocytes has not been prevented. Simultaneously, the Th1-type response plays a major role in vitiligo, accompanied by a large amount of IFN-γ. Ultimately, cytotoxic CD8^+^ T cells migrate to inflamed skin regions and induce apoptosis of melanocytes, leading to the depigmentation characteristic of vitiligo.

In conclusion, we highlighted the crucial role of the innate immune system in vitiligo (**Figure 4**). The activation of PRRs and release of DAMPs lead to increased secretion of inflammatory cytokines, promoting the activation and migration of immune cells like DCs and NK cells. The accumulation of ROS and the release of HSP70 induce oxidative stress, exacerbate innate immune activation, inflammation, and melanocyte apoptosis. Additionally, ER stress and ferroptosis not only directly cause cell death but also aggravate melanocyte destruction by recruiting inflammatory mediators and immune cells. Critically, these innate immune mechanisms converge to initiate and enhance adaptive immune responses, primarily through the activation and recruitment of autoreactive CD8^+^ T cells. Aberrant adaptive immunity is another key factor in vitiligo pathogenesis. Antigen presentation by DCs activates CD8^+^ T cells, which migrate to lesion sites and destroy melanocytes by secreting IFN-γ, perforin, and granzyme B. The imbalance between Th1, Th17, and Treg cells exacerbates the inflammatory environment. Furthermore, B cells contribute to vitiligo by secreting autoantibodies against melanocyte antigens and regulating T cell function. These findings provide new perspectives on how innate immune responses bridge to adaptive immunity in vitiligo, offering critical insights into disease progression. Therapeutic strategies targeting the interaction between innate immunity and CD8^+^ T cells hold promise for developing more effective and specific treatments for vitiligo.
